# Communication with children about parental bipolar disorder: a qualitative interview study

**DOI:** 10.1186/s40345-025-00384-9

**Published:** 2025-05-24

**Authors:** En-Nien Tu, Kate EA Saunders, Layla Rashid, Louise Dalton, Cathy Creswell

**Affiliations:** 1 Department of Psychiatry and Experimental Psychology, University of Oxford, Oxford, UK; 2 Chang Gung Memorial Hospital, Keelung, Taiwan; 3 Chang Gung University, Taoyuan, Taiwan; 4 Department of Psychiatry, University of Oxford, Oxford, UK; 5 Oxford Health NHS Foundation Trust, Warneford Hospital, Oxford, UK

**Keywords:** Depression, Mania, Mood, Children, Parents, Family, Disclosure, Conversation, Understanding, Relationship

## Abstract

**Background:**

The impacts of parental bipolar disorder (BD) on families and children highlight the need to understand how best to talk to children about their parents’ diagnosis, especially as their developmental capacity for understanding grows. This qualitative study aims to explore the strategies, challenges, and support needs of parents in relation to communicating with their children (5–12 years) about BD, in order to inform the development of further interventions and resources.

**Methodology:**

Purposive and snowball sampling strategies were used to recruit parents with BD, their partners, and stakeholders who support parents with BD. Recruitment occurred via social media, emails, and community outreach between April 2022 and April 2023. Semi-structured interviews were conducted with 11 parents with BD or non-BD partners and 12 charity workers or mental health professionals. The interview guides explored participants’ lived experiences and professional insights into communicating about parental BD with children. Data were analysed using reflexive, inductive, thematic analysis.

**Result:**

Participants identified several benefits of sharing parental BD diagnoses with children, including fostering understanding, adaptation, compassion, and strengthening family relationships. However, they also noted challenges such as uncertainty, stigma, and potential distress for children. To make communication effective, participants emphasised the importance of age-appropriate dialogue, addressing children’s concerns, providing reassurance, and preparing them for future episodes. They highlighted that transparent, interactive communication, thoughtful timing, and collaboration with family members and professionals are crucial for tailoring the process to each family’s unique needs.

**Conclusion:**

Our findings underscore the complexities of communicating a parental BD diagnosis to children, highlighting both the potential benefits and challenges. Participants emphasised the need for developing interventions and policies specifically tailored to address the particular communication needs of families impacted by BD.

**Supplementary Information:**

The online version contains supplementary material available at 10.1186/s40345-025-00384-9.

## Background

Bipolar disorder (BD) affects approximately 2% of the global population (Nierenberg et al. 2023) and can disrupt parental engagement, emotional regulation, and consistency in parenting practices (Tu et al. 2025). These disruptions also extend to family dynamics, with families affected by BD reporting less cohesion and more conflict (Stapp et al. 2020). Parent-child interactions, particularly parental neglect, negativity, and the extent to which parents provide structure and boundaries are key factors influencing children’s mental health outcomes and may be particular challenges in the context of parental BD (Doucette, 2016; Iacono, 2018; Serravalle, 2021). Moreover, the stress of parenting can intensify both personal and relational strain in parents with BD, negatively impacting upon their own mental health (Davison 2018; Tjoflåt 2012). Thus, supporting effective parenting practices and enhancing parent-child interactions are crucial for promoting mental health in both parents with BD and their offspring.

Effective communication is a key component of parent-child interactions, particularly in the context of chronic parental ill health, such as BD. As highlighted in a review on communicating life-threatening physical health conditions in parents (Dalton, 2019), this involves sharing factual information, acknowledging emotional responses, tailoring discussions to the child’s developmental level, and encouraging questions. However, qualitative studies with families in which a parent has BD suggest that the unpredictable nature of the disorder, coupled with a lack of open discussions at home, may lead children to develop misconceptions and experience emotional distress (Backer et al. 2017; Campos, 2018; Chen et al. 2021). Parents often grapple with how much to communicate about their experiences of BD, selectively sharing information according to their children’s age, emotional readiness, and the potential impact on family dynamics, challenges further compounded by the heightened risk of psychopathology and functional impairments among offspring (Chen et al. 2021, 2023; Tjoflåt, 2013). Environment stressors, such as disrupted family dynamics (Menculini, 2020), interact with genetic predispositions (Johansson, 2019) to create a complex gene-environmental risk that increases vulnerability to psychopathology in offspring of parents with BD. Longitudinal studies illustrate this risk across developmental stages: preschoolers exhibit elevated ADHD rates (Birmaher et al. 2021); as children reach school age, they begin to show increased anxiety, disruptive behaviour disorders, and major mood episodes (Axelson et al. 2015). Furthermore, lower global and social functioning becomes particularly prominent during adolescence—especially when accompanied by psychopathology (Helmink et al. 2023) and by middle adulthood, BD affects 11-13% with a 65% prevalence of lifetime mood disorder (Helmink et al. 2024). The context clearly introduces complexities for parents with BD regarding how best to communicate with their offspring about BD. However, research indicates that open communication about mental illness not only reduces children’s anxiety (Gladstone et al. 2011; Pihkala et al. 2012) but also alleviates parental stress (Rapa et al. 2024), potentially benefiting the mental health of both parties. These findings underscore the need for targeted research on how BD is communicated within families to inform interventions and guide policy decisions to better support these vulnerable families.

While research specifically focused on communicating about BD is scarce, valuable insights can be drawn from broader mental health contexts. For example, qualitative research with parents who have depression shows they often seek support to manage guilt, shame, and uncertainty regarding how to effectively communicate their condition (Pihkala 2008). Furthermore, a randomised controlled trial showed that the Family Talk Intervention (FTI), which facilitates parent-child conversations about parental depression, significantly reduced mood and anxiety symptoms in children, with effects lasting up to 1.5 years (Solantaus, 2010). Moreover, qualitative research showed that FTI helped children better understand their parents’ illness, reducing their worries and improving social interactions, while parents reported enhanced confidence and reduced feelings of shame and guilt (Pihkala 2012). These findings indicate that parent-child conversations about illness can have positive effects, making it crucial to identify the unique challenges faced by families with a parent who has BD and develop tailored support interventions.

Building on this foundation, the current study seeks to explore parents’ lived experiences of parental communication about their BD with their children, alongside perspectives from professionals working with parents with BD. Incorporating professional insights can potentially broaden and diversify the range of parenting experiences considered. The study’s objectives are to explore: (1) parents’ experiences and professionals’ observations of how BD is communicated to children; (2) the impacts of this communication on parenting practices, the parent-child relationship, and child mental health; and (3) the challenges or barriers parents face in communicating their BD to their children.

## Methods

### Participants and recruitment

Eligibility criteria were that individuals should be 18 years or older, residing in the UK, and fitting one of two categories: (1) Parents with BD or their partners, living with a child aged 5 years or older. The parent must have been diagnosed with BD before the child turned 12, with a focus on parenting experiences when the child was between 5 and 12 years old and had experienced anxiety problems during that period (due to the study being part of a broader research programme). (2) Stakeholders with professional experience in supporting a parent with BD who is the primary caregiver of a primary school-aged child.

To capture diverse perspectives on parenting with BD, we adopted purposive sampling to select participants across various categories of interest. For parents, we included individuals with varying roles (parents with BD and ‘well’ partners), family structures (single or dual parents), parental gender, education levels (O-level, A-level, undergraduate degree, postgraduate degree), and employment status (full-time, part-time, unemployed). We also considered the severity of parental BD, including age at onset (pediatric-onset or adulthood-onset), history of hospitalisation (yes or no), and time spent emotionally well since becoming a parent (> 70% or < 40%, estimated as the upper and lower thirds based on data from (Kupka, 2007)). For stakeholders, we aimed to include professionals from various sectors (charity, NHS, private practice), with diverse professional backgrounds (e.g., psychologists, social workers, psychiatrists, nurses), and from different regions of the UK (Scotland, Wales, Northern Ireland, North England, Midlands, South England; with a minimum representation of four regions).

Recruitment took place from April 2022 to April 2023 through social media (Facebook, X [formerly Twitter], and LinkedIn), emails, and printed advertisements distributed to voluntary and community organisations. Snowball sampling was also used, asking participants and organisations to share study information with others who might meet the inclusion criteria. Regular meetings between the first author (ET) and senior team members (KS, CC) were held to monitor participant diversity and determine when to cease recruitment. 15 parents and 18 stakeholders expressed interest in participating in the study. Of these, two stakeholders were ineligible, three parents and four stakeholders did not respond, and one parent withdrew. Ultimately, 11 parents and 12 stakeholders participated in the study.

### Data collection

We developed a semi-structured interview guide (detailed in Online Supplement 1) using the following process: an initial draft was created based on a review of prior literature, e.g., Dalton (2019), clinical experience, and our study objectives. The draft was refined through discussion meetings with our research team to ensure comprehensive coverage of key issues. Finally, the guide was pilot-tested and further revised with input from Public and Patient Involvement (PPI) contributors with relevant lived experience to confirm the feasibility and relevance of the topics and questions. The guide included main questions, follow-ups, and prompts for both parents and professionals. For parents, topics covered how they discuss their mental health with their children, the challenges they faced, the impact on parenting and the parent-child relationship, and their children’s adjustment. The professionals’ guide explored their experiences in supporting parents with BD in communicating with children about the condition, along with the associated concerns and challenges.

To ensure informed consent, participants submitted a written consent statement via email before the interviews. At the start of each interview, the interviewer reviewed the explanatory statement, addressed any questions, and obtained the verbal consent. A safeguarding protocol was in place to manage potential distress, involving pausing the interview, assessing well-being, consulting the safeguarding lead, and directing participants to their General Practitioner (GP) or mental health resources if necessary. However, the protocol was not needed in any interviews. The first author conducted all interviews, with most including a co-interviewer (a graduate student or research assistant). The first author transcribed all interviews verbatim, with either LR or EH reviewing and correcting uncertain segments as needed. Researchers also took memos during and after each interview for documentation and further analysis. The team held regular meetings to debrief, discuss the interview process, and consider emerging themes.

### Data analysis

We used reflexive, inductive thematic analysis, as proposed by Braun and Clarke (2021), to generate codes and themes, identifying patterns of shared meaning. Rooted in an essentialist/realist framework, our analysis focused on capturing the lived experiences and support needs of parents with BD, as driven by the dataset itself.

ET immersed himself in the data by replaying recordings and transcribing dialogues, ensuring confidentiality by removing all identifiable information. NVivo, Version 12, was employed for data organisation and coding. ET performed initial line-by-line coding, considering the context and participants’ overall views, which were enhanced by field notes. EH and LR then reviewed the transcripts, providing feedback and refining codes to ensure thorough analysis. Subsequently, the codes were grouped by thematic significance, undergoing multiple iterations of review and refinement until distinct themes emerged. The wider research team (LR, LD, KS, CC) provided critical feedback to refine codes and themes, ensuring only relevant data were integrated.

Instead of data saturation, we adopted the concept of “information power” (Malterud 2016), which emphasises collecting data that is rich and relevant enough to address the research questions. By gathering diverse insights, we ensured the data comprehensively covered core issues without requiring an exhaustive exploration of all themes. Information power was assessed based on sample specificity, dialogue quality, and alignment with the study’s aims, ensuring sufficient depth for drawing robust conclusions. The first author, in consultation with CC and KS, determined the appropriate end-point for data collection.

### Reflexivity statement

Our research team is culturally and professionally diverse, but united by a shared focus on the effects of parental BD on children. Both professional expertise and personal experiences as parents inform our thematic analysis. The lead author, a psychiatrist with personal ties to BD, draws on experiences facilitating family conversations in healthcare and domestic settings. The team includes a doctoral candidate studying children from refugee backgrounds, adding insights into the intersection of mental health and social marginalisation, as well as experts in mood disorders and child psychotherapy. Our parenting experiences, from infancy to adulthood, as mothers (CC, LD) and fathers (ET), and varied cultural perspectives—East Asian, Middle Eastern, and Western—enhance our interpretive framework. Regular reflective discussions and notes helped balance and integrate diverse viewpoints, allowing us to capture the complex dynamics within families affected by BD and ground our research in a comprehensive and empathetic narrative.

### Ethical considerations

The Medical Sciences Interdivisional Research Ethics Committee at the University of Oxford approved the study (Ethics Approval Reference: R76164/RE007). We recruited three service users (two parents with BD and one partner of a parent with BD) to be PPI contributors, who reviewed our research process and materials, including advertisements, the participant information sheet, the assessment form, and the topic guide.

**Table 1:** Parent demographics (*n* = 11)

Demographics	Number of Participants
Mother with BD	5
Father with BD	3
Partner of mother with BD	1
Partner of father with BD	2
Age of parent who attend the interview	
• 30–39	3
• 40–49	4
• 50–59	3
• 60–69	1
Age of children at the time of interview	
• 5–8	3
• 9–12	8
• 13–19	5
• > 19	2
Child living with	
Both parents	9
Single parent	2
Ethnic background	
White	11
Non-White	0
Highest level of education	
Graduate	8
Undergraduate	1
A-level	0
O-level	1
Prefer not to say	1
Employment status	
Full-time job	5
Part-time job	2
Unemployed	4
UK region	
South England	9
• East	2
• London	2
• Southwest	1
• Southeast	4
North England	1
Northern Ireland	1
Age of onset of parental BD	
10–19	4
20–29	5
30–39	2
Past psychiatric hospitalisation	
Yes	4
No	7
Percentage of time being well since having the child	
Up to 40%	2
41–60%	3
61–80%	3
81–100%	3

**Table 2:** Stakeholder demographics (*n* = 12)

Demographics	Number of Participants
Charity volunteer	3
Charity worker	2
Social worker	2
Clinical psychologist	1
Family therapist	1
Mental health nurse	1
Psychiatrist	2
Age of Stakeholder	
• 20–29	2
• 30–39	
• 40–49	2
• 50–59	5
• 60–69	3
Gender of Stakeholder	
• Female	12
• Male	0
UK region	
Scotland	3
North England	1
Midlands of England	2
South England	5
Wales	0
Northern Ireland	1

## Results

### Study characteristics

We interviewed 8 parents diagnosed with BD (5 mothers and 3 fathers), and 3 non-bipolar partners or ex-partners of parents with BD (1 father and 2 mothers from separate households). As shown in Tables 1 and 8 of 11 parents held graduate degrees, with most co-parenting in the same household (9 out of 11), predominantly from Southern England (7 out of 11), and all identified as white. Stakeholder participants, detailed in Table 2, included 5 charity workers or volunteers and 7 mental health professionals or social workers, providing diverse insights into supporting families affected by BD. Interviews were held via Microsoft Teams (*n* = 21) or telephone (*n* = 1), audio-recorded except for one parent who, due to mood, responded via written questions on Qualtrics. The average interview duration was 86 min for stakeholders (range: 52 to 103 min) and 95 min for parents (range: 45 to 154 min), including two parent interviews split into two sessions due to scheduling constraints. No adverse events or distressed occurred during discussions of BD communication.

Our analysis generated 74 refined codes—52 from both parents and stakeholders, 9 from parents only, and 13 from stakeholders only—distributed across themes: Theme 1 (Perceived Advantages) with 10 codes, Theme 2 (Perceived Challenges) with 11 codes, Theme 3 (Key Elements) with 29 codes, and Theme 4 (Practical Strategies) with 24 codes, with subtheme details in Supplement 2. The theme structure is robustly supported by triangulation, with most subthemes (8 of 12) incorporating perspectives from all five roles—fathers and mothers with BD, non-bipolar partners, mental health professionals or social workers, and charity workers—enhancing the findings’ reliability, while all subthemes include contributions from at least four roles.

### Theme 1: perceived advantages of communication about BD

Participants highlighted several benefits of discussing parental BD, emphasising both educational and relational aspects. This communication was viewed as enhancing children’s awareness and understanding of BD, promoting their personal growth, and strengthening trust and emotional bonds within the family. These perceived advantages are explored through the following subthemes.

#### Subtheme 1.1: promoting understanding and reducing misconceptions in children

Parents and professionals noted their beliefs that children often form their own interpretations of parental BD, which can lead to misconceptions and fear. A father with BD explained: “*They [children]… have all sorts of theories [about parental BD]… until they’re told the reality*” (P07). A mental health nurse shared an example from their experiences, suggesting that without accurate information, children may become anxious and confused when trying to make sense of their parents’ unpredictable behaviours: “*The children don’t actually know what’s happening [during an episode]… then they become frightened and scared of what the parents are actually doing and trying to conceptualise it for themselves*” (S08).

Parents and professionals both reported that effective communication can help children understand how BD affects parental behaviour and reduces misconceptions. A family therapist noted: “*It [communication] is fair to both parties… because when the child is more aware… why is my mum or my dad saying something like this? They don’t mean to say something like this*” (S01). A charity worker highlighted that honesty reduces anxiety: “*It’s much better to be honest (about parental BD)*,* because then it’s not so frightening for the child*” (S07). This understanding can also empower children to cope with parental mood swings and may help them avoid blaming themselves for their parents’ behaviours. As a mother with BD shared: “*Often she [daughter] goes ‘it doesn’t matter… I know it’s just your mood.’… she knows it is just my illness*” (P08). Furthermore, sharing information about BD may foster psychological maturity, encouraging children to reflect on their interactions with their parent with BD. Another mother with BD described how this communication enhanced her child’s empathy: “*She [the child] understands everybody’s individuality… she understands some of [others’] things are not her things. So*,* seeing it from their perspective… I do feel like [this understanding] made them more compassionate towards not just me but everyone*” (P06).

#### Subtheme 1.2: fostering expression and strengthening bonds in children

Parents and professionals observed that discussing BD can encourage emotional dialogue between parents and children, particularly since children may hesitate to share their emotions for fear of upsetting their parents with BD. As one mother with BD remarked: “*Sometimes he [my son] doesn’t always want to say how he feels because he is worried that he’s going to affect you or upset you*” (P04). A mother with BD shared her belief that fostering open communication creates a safe environment for both parents and children to express their concerns, noting that such transparency not only “*gives [children] the opportunity to come and talk to me or their dad if they have anxiety over something in their lives*,” but also makes it “*much easier to tell them if I am in a depressive episode”* (P11). A peer-support group facilitator explained that this approach helps children articulate their emotions: “*They [parents with BD] would describe how they feel… they got their kids used to talking about their feelings in this way and started explaining… ‘This is how I feel today’”* (S04). Professionals specifically observed that engaging in honest and supportive dialogue can also help reduce feelings of isolation, as children may feel they are the only ones who have a parent with BD. A charity worker observed: “*Children in this situation feel shame… because mental illness isn’t talked about*,* and they think that they’re the only ones experiencing family life like this*” (S02).

Parents and professionals also reported that discussing BD with children can strengthen the parent-child bond and build trust. A father with BD affirmed: “*I think [talking about] it is a good thing… it definitely does help the bond because we’re able to talk about that [parental BD]*” (P05). This communication may also help maintain connections in families experiencing physical separation. A peer support group facilitator recalled how one father, living apart from his daughter, used letters to bridge their communication gap: “*His daughter [initially] refused to talk to him… he found it easier to communicate [through letters]*,* explaining to her… why he’s not together with her mum*,* why he sometimes acts the way he does*,* and how he feels… I know they did exchange a few letters and he found a lot of comfort in them*” (S05). However, it remains unclear how the daughter perceived these letters or what impact they had on her.

### Theme 2: perceived challenges of communication about BD

Participants voiced concerns about communicating parental BD to children, particularly regarding uncertainties around what to share, when to share, and how to approach the discussion. These challenges were further complicated by fears of how children might perceive the conversation and the psychological burden it could impose. This underscores the need for thoughtful guidance to help parents discuss BD in a way that maximises benefits while minimising harm, as explored in this theme.

#### Subtheme 2.1: fears of placing emotional burdens on children

Both parents and professionals expressed concerns that sharing a parent’s BD diagnosis could confuse or distress children particularly if the conversation isn’t tailored to their age and emotional readiness. A charity worker emphasised the need for sensitivity in this process: “*The child could be really upset at the end of [the conversation] or not want to take the information in. I think that depends on that child and parent relationship and how it’s sensitively conveyed*” (S03). This risk became evident when one daughter appeared overwhelmed by her mother’s unplanned disclosure of past suicidal thoughts: “*I was just waiting at the bus stop*,* and I said something about when I was suicidal and [my daughter] went ‘What?’ I hadn’t realised I hadn’t mentioned it… she was really*,* really shocked… oh bless her”* (P08). Worries about triggering such emotional impact may heighten parental hesitation in starting the discussion. A father with BD explained: “*I would not go into the ins and outs of what bipolar is… it could easily frighten them [the children]*” (P07). Concerns about hereditary risks also weighed heavily on some participants. A mother with BD worried: “*I would be really concerned that he [my son] might worry that he might have it [BD] and get diagnosed with a mental illness too*” (P02). She also feared her postpartum diagnosis might emotionally burden her son: “*I’m really concerned because of the way I was diagnosed as being a postpartum-related diagnosis*,* I am really concerned that [the son might] ever feel that it was his fault*” (P02). Professionals specifically observed that these conversations can become more complex when both the parent and child have mental health concerns. A peer group facilitator noted: “*If you’re in that situation of a parent having bipolar and their child having anxiety problems*,* you wouldn’t want to be a burden on the other. So often*,* you would not communicate what’s going on because you know that the other one is struggling*” (S05).

#### Subtheme 2.2: self-acceptance and stigma as barriers to communication

Both parents and professionals suggested that the way parents perceive their diagnosis influences their willingness to communicate about it. A social worker observed, “*The parent’s relationship to the diagnosis… if a parent doesn’t find that diagnosis particularly helpful or they’re feeling stigmatised by the diagnosis… that informs how they might want to talk about their mental health with their children”* (S09). Struggles with self-acceptance can limit openness, as a charity worker explained: “*[Disclosure] is sort of like how parents feel comfortable about their own diagnosis if they accept it” (S03).* Participants noted that when parents do not feel the BD diagnosis is helpful or do not recognise it, they may avoid communication altogether. One mother shared that her ex-partner refused to talk about his BD: “*In terms of my kids*,* they have a dad that will not talk about his bipolar. It does not come up in conversation ever. He actively avoids it… it often leads to real disappointment [in children]*” (P03).

Both parents and professionals expressed concern about how children might perceive their parents after discussing parental BD. Parents worried their children would see them as “*something wrong*” (P06), “*seriously different from other dads*” (P03), *“weird*” (S10), or “*not capable of looking after [their children]*” (S07). These fears could shift family dynamics, leading children to view the parent with BD as less reliable. A “well” father observed: “*It [the conversation] made them [the children] very much more dependent on me. They felt that… I was the safer parent to be with*” (P10). Some parents also feared their children’s reactions, as one mother with BD shared: “*I haven’t really explored it [parental BD] with him [the child]… I’m a bit scared to ask him directly. You know how you feel scared of what his answer would be*” (P02). Professionals noted that stigma could also extend to interactions with extended family and the community, further complicating these conversations. A social worker shared their experience of working with parents who perceived that “*If I [the parent] tell my kids*,* then my kids are going to tell the neighbours’ kids*,* which means the neighbours know*,* and I think it’s all wrapped up in stigma*” (S10).

#### Subtheme 2.3: uncertainty in communicating about BD

Both parents and professionals highlighted the uncertainty that parents face regarding what, when, and how to communicate about BD. A psychiatrist noted that parents often question: “*How much do I tell them [children]? What do I share? How do I share?… Am I in some way contributing to their anxiety by either showing them too many of my symptoms or hiding my symptoms?*” (S06). The unpredictable course of BD symptoms can make it especially challenging to answer questions like “*Are you always going to be like this?*” (P05), “*Why are you ill?*” (S06), or “*Is this going to happen to me?*” (P07). As a father with BD explained, the episodic nature of the disorder can complicate these conversations: “*It’s a bit difficult to explain just how it’s sometimes… most of the time I’m okay*,* and then sometimes I’m not*” (P05). A charity worker added, “*The biggest struggle in the conversation [with children] was to talk about the fact that bipolar disorder cannot be fixed*” (S04). Determining the right time to share information about BD is also challenging. A charity worker noted parents with BD often ask, “*When they should tell [their children]*” (S07). The child’s age and developmental stage seem to play a significant role, as one mother described: “*When they [the children] were very little*,* it was really difficult to explain that [parental BD] to them to make any sense*” (P03). As children grow older, parents may fear their child’s increasing awareness and deeper questions, as another charity worker observed: “*[Children are] asking more questions [about parental BD] and starting to become more aware*” (S03). Parents often grapple with uncertainties about BD’s heritability, as a mother with BD shared: “*I think nobody really knows what the genetic links are*” (P02).

### Theme 3: key elements of communication about BD

Participant accounts identified four key elements of communication about BD: tailored communication, addressing children’s concerns, providing reassurance of a consistent personality and love, and preparing for future episodes. These insights are derived from participants’ lived experiences and their beliefs about what contributes to the communication process with children.

#### Subtheme 3.1: age-adaptive communication

Both parents and professionals noted that discussions about BD often begin with issues relevant to children, such as changes within the family. One mother explained: “*It started off as a conversation [with the children] about why we are living here? Why aren’t we still living with Daddy [who has BD]? So we first kind of tackled that… to allow her [the child] a little bit of understanding about what bipolar was… why he behaved in the way that he behaved at the time*” (P03). For younger children, participants emphasised using simple, everyday language, avoiding medical terms like “*psychosis*” (S08), “*neurochemicals and neurotransmitters*” (S12), and “*brain chemical imbalance*” (P07). Instead, parents recommended phrases such as “*I have something in my brain*” (P04), “*my brain doesn’t work the same*” (P06), or “*Papa goes up and down with his mood*” (P09). As children mature, more detailed information can be introduced through online articles (P03), scientific explanations (P07), or websites about BD (P09). Participants suggested that using familiar analogies or metaphors may help explain BD to children. One mother compared the disorder and its treatment to a physical injury: “*It never goes away because once you’ve broken a bone*,* it remains weaker than it was before… It’s like when you break your leg*,* you have to have medicine to manage the pain… wear a cast… to make it better*” (P03). To make these explanations resonate, parents sometimes develop unique terms that fit their own context, referring to medications as “*my crazy pills*” (P06) or using “*sleep diarrhoea*” for excessive sleeping (P10), reflecting how they personalise language to better communicate with their children.

Both parents and professionals emphasised the importance of developmentally appropriate, child-friendly materials for communicating about BD. For younger audiences, a psychiatrist suggested using familiar cartoon characters: “*I think it’s got to be done in their language… [like] Winnie the Pooh… if you look into it*,* all the characters have a mental health issue… that’s the way to present it to very young children”* (S12). Storybooks were also valued for age-appropriate dialogue, with a charity worker noting: “*We’re sending a resource to somebody that could sit down and have a conversation in a book with the child through a story*” (S03). Mood charts were another tool to engage families: “*You’re looking at the sort of zero being the really low mood*,* suicidal*,* to the mania*,* the psychotic… the ten… they could use it alongside the family and say*,* like*,* ‘What’s your mood today?’… It could become a resource that everybody on the fridge has a mood scan*,* ticks their mood each day to see how’s everybody doing*” (S03). Participants also advocated for simple, practical aids like leaflets to ease the burden on parents. A peer-support group facilitator shared: “*A simple tool like a little leaflet… takes the pressure off the parents… [on] how to explain it [BD] to a child*” (S04). Engaging visuals were emphasised, as a nurse explained: “*Something like that with lots of pictures… not like diagrams… because that then gets a bit too anxiety-provoking*” (S08). However, participants acknowledged a shortage of resources for younger children. A social worker pointed out: “*Books that you tend to have are written for teenagers… there’s nothing out there for littlies that says… sometimes mum might be like this*” (S10).

#### Subtheme 3.2: addressing children’s concerns and misconceptions

Both parents and professionals highlighted that clear, two-way dialogues may help children manage emotions like worry, guilt, and the caregiving burden associated with parental BD. Open communication was seen as key to preventing children from feeling responsible for their parents’ condition. A father with BD stated: “*I always try to say that it’s not about you. I’m not depressed because of you. And that it’s not their fault*,* so [I’m] trying to explain the triggers without making them worry about me or blaming themselves*” (P05). Addressing children’s fears about the severity of BD was also perceived as crucial by a charity worker: “*They [the children] look up bipolar and see it’s got the highest suicide rate of any condition… they might think you’re going to die. Every time you go out*,* they might be worried about you… I think you need to face those things and decide how you are going to do it… in a very thorough way*” (S07). One father discussed clarifying misunderstandings, such as confusing physical illness with BD: “*The slightest hint of illness will make her [the child] very worried… if [the mother with BD] has COVID and is in bed for a day*,* that associates with ‘Mummy’s really depressed’*,” said another father (P10). He also addressed children’s fears about inheriting BD: “*[My child] thinks that if I don’t sleep well for a night*,* I might end up… being carted off into hospital against my will… I explained that if you have an illness called bipolar*,* and then you go without sleep*,* it can make you look like this [relapse]… if someone who doesn’t have that condition [BD] goes without sleep*,* they don’t have any problems*” (P10). Discussing genetic risks requires reassurance, as a charity worker added: “*It’s important to make that clear… you’re probably not going to have this [BD]*,* but even if that was the case*,* we as a family know an awful lot about it*,* we can support you*” (S07).

#### Subtheme 3.3: reassuring consistent love and personality

Parents and professionals both highlighted the importance of instilling hope in children by emphasising that BD episodes are temporary and treatable. One mother explained that her son needs “*someone to assure him that… it will get better… the situation*,* or the father [with BD]*” (P09). Participants emphasised the consistent nature of parental love, as a social worker explained: “*Mum’s got something going a bit kooky in her mind… it makes her do some strange things sometimes*,* [but it] doesn’t stop her loving you*” (S10). Showing that parents continue to make efforts, even during BD episodes, may reinforce this message. A peer support group facilitator remarked: “*Even when someone is down*,* they are there for you just in their own way and in the best that they can do that day*” (S05).

Reassuring children about the continuity of personality amid BD’s impacts was perceived as essential by parents. One father observed how children may struggle to distinguish between their parent’s normal emotions and those tied to BD: “*If she [the mother with BD] gets a little excited… feels a bit down*,* or gets cross*,* there will be three people in the family thinking*,* ‘Oh*,* is this the start of an illness episode?*’” (P10). This blurred line between typical behaviour and BD symptoms can be confusing for children. A mother suggested helping children “*recognise which elements of him [the father with BD] are driven by the bipolar and which elements of him are just him*” (P03). Another mother used a nickname for her BD: “*I call my bipolar Betty… it’s separate from me… a part of me*,* but she’s not who I am. It just made more sense to explain it to a child in that way… it’s not his mum. I’ll just say*,* I feel like I’m Betty. Betty’s really like taking over at the minute*” (P04). This approach might help children understand that while BD affects their parent, it doesn’t define who they are.

#### Subtheme 3.4: preparing and supporting children for future BD episodes

Given BD’s episodic nature, both parents and professionals stressed the importance of preparing children for future episodes to foster resilience and provide a sense of control over the situation. One father with BD explained how he prepared his son for potential mood changes: “*I explained to him [the son] what might happen… make sure he’s informed about how I feel and then say to him… don’t worry about it too much if I react this way*,* which is my illness [BD]*” (P01). A charity worker advocated for proactive planning, encouraging parents to think through difficult scenarios and explain these plans to their children: “*Think through scenarios that you don’t want to think about… if I go into hospital*,* what happens to my child… If it’s actually written down*,* and you explain it to them… children are not going to be so worried*” (S07). A mother with BD further highlighted the need for maintaining appropriate boundaries, stating that children’s role should be supportive, not caregiving, when parents are unwell: “*I never want my children to feel it’s their job to look after me*” (P11).

Parents acknowledged the value of helping their children understand their emotional needs during BD episodes. A mother sought to help her children recognise and interpret their father’s needs during his BD episodes: *“They need to learn to understand… when he [father with BD] is unwell… much more kind of extreme. It’s basically him saying… ‘I’m unwell*,* I don’t want to be bothered by you*,* leave me alone and let me be’*,* so it’s about recognising”* (P03). However, a father with BD cautioned against portraying the disorder to children as something uncontrollable, urging parents to avoid “*over-medicalising it*,* like*,* this is something that just happens out there*,* nothing can be done”* (P05). Instead, he emphasised the importance of expressing his emotional needs during an episode and encouraging his children to offer simple forms of support without making them feel responsible for his care: “ *It’s just giving me a cuddle*,* or… encouraging me to come out*,* or do something fun when I’m down… how the child can help you… [so they] doesn’t feel like you have to look after and end up being a carer… almost like a relapse prevention plan for both of you… ‘If daddy’s unwell*,* I can do this’*” (P05).

### Theme 4: principles of communication about BD

This theme explores strategic principles that parents with BD may apply to communicate effectively with their children. It focuses on determining the optimal timing, selecting the appropriate level of detail, and adopting a clear and interactive communication style. The theme also examines the roles that family members and professionals can play in facilitating these crucial discussions.

#### Subtheme 4.1: ‘how to communicate’ - transparency and two-way conversation

Both parents and professionals recognised that parents sometimes use oversimplified or vague language when sharing information about BD with children due to uncertainties about how much to share. However, they also recognised that children can sense when something is wrong, and incomplete communication may raise anxiety. A psychiatrist explained: “*They [children] obviously understand something is wrong… not telling them what exactly it is*,* is just going to raise anxieties*” (S12). Some parents, who had not understood their own parents’ illness, felt a strong motivation to be transparent with their children. One mother with BD shared: “*I grew up with my mum who had schizophrenia and I*,* in some ways*,* didn’t understand her illness. So I used to look after her and felt sad for her but not fully understanding why. I never want my children to feel this way*” (P11). Participants emphasised that while honesty is important, information should be delivered with sensitivity to the child’s emotional readiness and level of understanding. A charity worker noted: “*I think usually honesty is the best policy*,* but sometimes it’s about finding the right time… finding the right words*,* and answering questions and being available… just to make things as non-threatening as possible… they need to understand what it is they might have to deal with*” (S02).

Parents and professionals also highlighted the value of fostering a two-way conversation in which children feel safe enough to ask questions. A social worker noted: “*I think it’s not just the description of what the mental health presentation is; I think it is what the parent enables in the relationship—that you can talk to me. Even though these are difficult questions*,* I’m here and I’m able to listen… there’s something quite reassuring in knowing that I’ve got this worry*,* I’ve got this concern*,* and if I say it out loud*,* my parent is going to help me sort it out and give me back some information that is reassuring… The child is able to know that it’s safe to talk about their feelings and have them validated*” (S09). Participants observed that children often have questions about their parents’ BD, and it’s important for parents to address these concerns. One mother with BD shared: “*They [children] have asked about my medication*,* like*,* ‘What does it do?’… And they do ask… why sometimes I do certain things and why sometimes I don’t*” (P06). A father whose partner has BD added: “*He [the child] asks very probing questions and just keeps going with the questions… you’ve kind of got to go with what they feel they need*” (P10). Although parents might worry about providing the wrong answers or whether their children are ready for certain information, one mother shared her approach: “*Have the conversation led by the children*,* and just answer to the best of my ability*,* and try not to be scared of answering difficult questions… If you’ve asked the question*,* then you’re old enough to listen to an answer*” (P03).

#### Subtheme 4.2: ‘when to Communicate’– Timing and context consideration

Parents and professionals had varied opinions on when to communicate about BD, often influenced by the child’s ability to understand. One father with BD noted: “*Being 12 and 10*,* they would have a fairly reasonable amount of understanding”* (P07). Some parents waited for signs of maturity, as a charity worker explained: “*What age should I tell them… I think that’s very individual… one [child] can be very mature at five or six*,* and another might not be until they’re 10*” (S07). Others waited until BD symptoms became noticeable, with a mother with BD stating: “*I now discuss bipolar quite openly with my children as it has an impact on everyone*,* not just me*” (P11). Conversely, some participants felt that adults often underestimate children’s understanding. A psychiatrist shared: “*Children often know more [about parental BD] than you think they know” (S06).* As a result, some parents favoured early communication, tailoring explanations to the child’s level. One mother with BD likened it to sex education, saying: “*They [children] are never too young*,* but there’s always a level you can explain it at*” (P08). A partner of a father with BD added: “*I would not put an age limit on it [communication]… to avoid a surprise moment later in life”* (P09).

Parents and professionals identified emotional stability, preparation, and natural opportunities as key factors for effective communication. A social worker highlighted the need for stable moments for safe discussions: “*The challenge is if they [parents with BD] are in a high phase or a low phase*,* actually that pressure on them*,* when you’re trying to make somebody else feel better about what’s going on for you*,* it’s impossible… [Disclosure is] really triggering anxiety if… they’re trying to talk to you about what’s going on for them*” (S10). Careful planning was also recommended, with a charity worker suggesting: “*I think an important thing is to put a lot of thought into it*,* you know*,* to really think about it and have enough time to really sit down and have a long [conversation]… Plan in your head how you can say reassuring things… like… doing an advance statement or directive*” (S07). Participants noted that natural occasions, like media or daily interactions, could ease the conversation. A charity worker shared: “*We’d seen the character on EastEnders*,* and they [children] said… ‘What’s the matter with her [the character with BD]?’ and we talk about it [BD]… seeing them on the screen gives you an opportunity to bring the subject up*,” one charity worker observed (S07). Schoolwork also provided openings for discussions, as one father with BD noted: “*[Children’s] homework diaries had the symptoms of depression… so we talked about it*” (P05). A mother with BD used her academic assignments to educate her children: “*I practised [a BD presentation] with my two kids… It was ‘Can you help me with my schoolwork?’*,* but also giving them that information at the same time*” (P06).

#### Subtheme 4.3: ‘who should communicate’– family and professional support

Involving other family members in sharing information about BD was seen by parents and professionals as important but challenging. Some participants preferred to explain the condition jointly with their partner. One father with BD shared: “*We would have sat down together*,* my wife and I*,* and explained the whole thing*” (P07). During crises, participants noted that “well” parents often took the lead in providing explanations, though they faced the urgency and difficulty of these situations. A father whose partner has BD described: “*Suddenly it’s [mood episode] all happening*,* and I’m having to explain what is happening rather than being able to take my time explaining it in small chunks… before I’d been able to remove them [the children]*,* they’d seen some really upsetting stuff*,* and I had to explain that in the context*,* it was really hard*” (P10). How family members frame a BD episode can shape a child’s understanding and emotional response. A psychiatrist noted: “*How they [children] react would depend on the people around them. If they have a sympathetic relative who explains that mummy is really unwell… versus somebody saying*,* ‘Oh*,* mummy was a bad person*,* she’s been taken away’… So it does depend on the extended family around them*” (S12).

Professionals shared their experiences in assisting parents with BD to communicate their condition to their children, either by helping them prepare or guiding them during the conversation. One social worker explained: *“[Helping parents] think through their fears… and have them heard and validated so that they can then do it for their children”* (S09). Some professionals took the lead in communicating with children, similar to in psychoeducation sessions. A social worker described initiating these talks: “*I can pop you [children] some appointments… let’s sit them down… What do you want to know*,* how can we make this open and inclusive?*” (S10) Participants noted that professional involvement varied based on the child’s circumstances and the parents’ level of willingness. The social worker emphasised the need for flexibility: *“Depending on… the age of the child*,* the demographics… the ability of the child to understand*,* and how much the parent is involved or wants you [the therapist] to be involved*” (S10). She further stressed the importance of coordinating between adult and child mental health professionals: “*There might already be a team around the child… let’s go into this [conversation] together… a trusted children’s colleague and me*” (S10).

### Comparative analysis of thematic contributions

While all subthemes incorporate perspectives from diverse roles, a comparative analysis of parents’ (parents with BD and non-bipolar partners) and stakeholders’ (professionals and charity workers) accounts highlights both shared and distinct perspectives on communicating about BD with children. Parents uniquely emphasised leveraging personal and familial experiences to tailor communication strategies, evident in their use of personalised language for age-adaptive communication (Subtheme 3.1), efforts to help children distinguish personality and typical behaviours from BD while reinforcing continuity (Subtheme 3.3), insights into future episode preparedness by avoiding overmedicalization, expressing emotional needs, and empowering children to help (Subtheme 3.4), and motivation driven by their own childhood experiences of non-transparent parental mental health communication to foster openness with their children (Subtheme 4.1). Among parents, non-bipolar partners did not tend to refer to communication advantages and challenges (Themes 1 and 2), likely prioritising practical strategies and support (Themes 3 and 4) due to their caregiving role for children and partners with BD. Conversely, stakeholders focused on the systemic and external factors that influence communication, highlighting the importance of support systems and social dynamics in addressing emotional and practical challenges, as exemplified in their recognition of honest dialogue to reduce children’s isolation (Subtheme 1.2), identification of complexities when both parent and child have mental health issues (Subtheme 2.1), acknowledgment of community stigma as a barrier (Subtheme 2.2), and emphasis on professional involvement, flexibility, and coordination in facilitating communication (Subtheme 4.3). However, most codes reflected the views of both groups, and the overall thematic structure were not dependent upon the small number of unique perspectives that were shared. This synthesis underscores the complementary nature of parents’ experiential insights and stakeholders’ systemic perspectives, enriching the thematic structure.

**Fig. 1:**
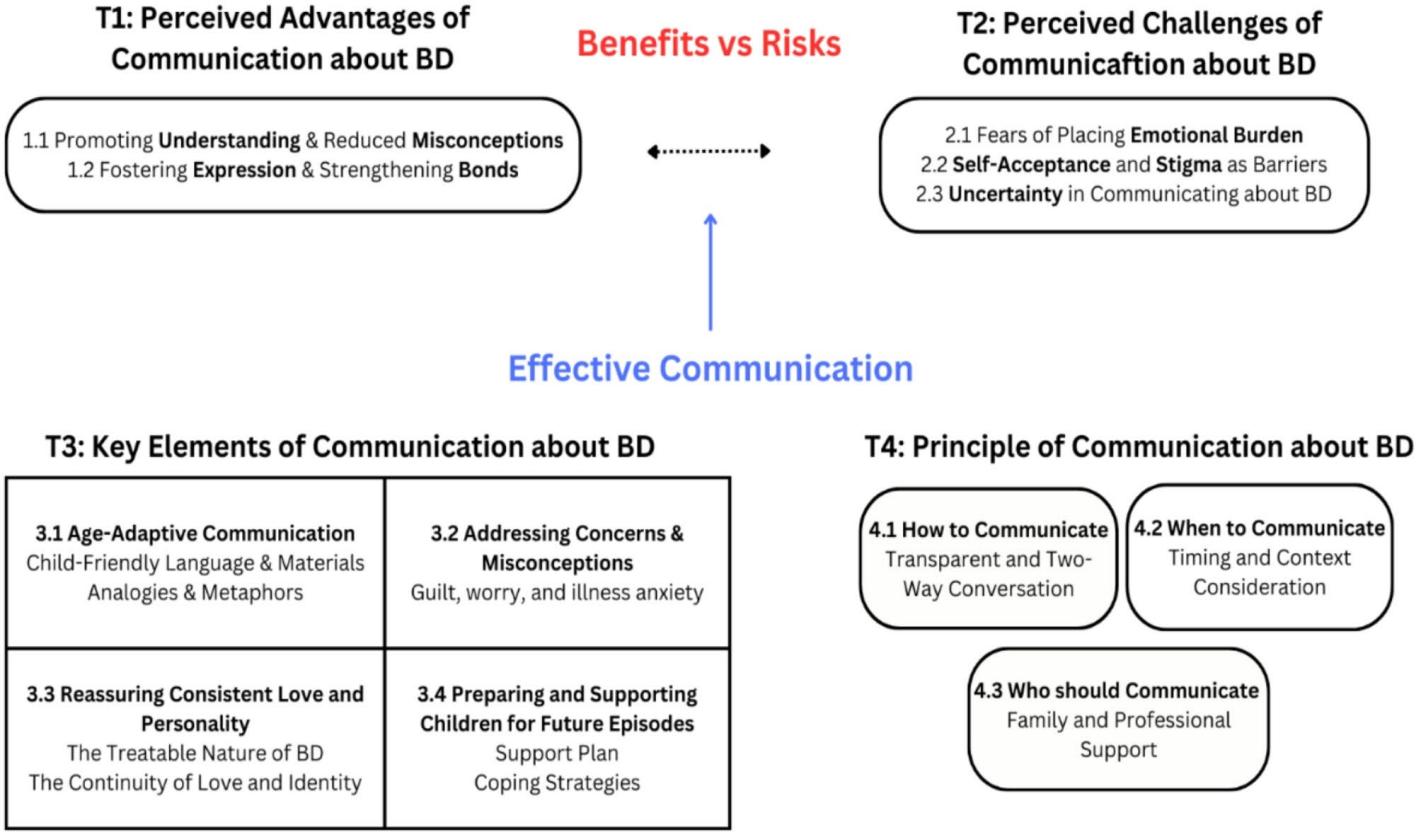
The conceptual model depicting themes and sub-themes

## Discussion

This study explored how parents with BD communicate with their children about their condition, drawing from the experiences and perspectives of parents with BD, their partners, charity workers, and mental health professionals. Through thematic analysis, four themes were generated, reflecting the complexities of this communication process (Fig. 1*illustrates the coding tree and relationships between themes and subthemes*). Participants shared that openly discussing BD with children had benefits, such as improving children’s understanding, reducing anxiety, and strengthening family bonds as well as trust. However, they also noted significant challenges, including struggles with self-acceptance, fears of being judged, and uncertainties about how much to share while protecting their children from distress. Participants emphasised the importance of using age-appropriate language and materials, addressing concerns and misconceptions, reassuring children of their parent’s consistent love and identity, and preparing them for future episodes with coping strategies and support networks. Transparent, interactive communication, thoughtful timing, and collaboration with family members and professionals were highlighted as key to tailoring the process to each family’s needs. These insights offer a practical roadmap for navigating the complexities of sharing parental BD, providing essential guidance for developing supportive resources to facilitate these conversations effectively.

Communicating a parental diagnosis of BD to children involves balancing potential benefits and risks. This communication was perceived to provide emotional relief by helping children better understand their parent’s condition (Subtheme 1.1), but concerns were also raised that the emotional burden might overwhelm them (Subtheme 2.1). Some participants noted that effective communication strengthened parent-child bonds (Subtheme 1.2), while others expressed worries that it could negatively affect children’s perceptions of their parental roles (Subtheme 2.2). These contrasting experiences underscore the need for more research on the emotional and relational impacts of these conversations. While not specifically designed to guide parents in discussing BD with children, Family-focused therapy for high-risk youth (FFT-HR)—for those with subthreshold BD symptoms and a family history—offers therapist-supported family conversations on mood symptoms, communication, and problem solving to enhance understanding and coping. Randomised controlled trials show that FFT-HR improves mood symptoms and delays mood episode relapses (Miklowitz et al. 2013, 2020), with Miklowitz and Chung (2016) emphasising open family dialogue as a potential key factor, particularly among families with high-expressed emotion, where it may counter criticism and hostility. Furthermore, Family Talk Intervention, which helps parents communicate about depression, has been found to significantly improve children’s mood and anxiety symptoms (Solantaus, 2010). Likewise, interventions for families facing parental HIV and cancer have shown positive effects on children’s emotional well-being (Betancourt, 2017; Murphy, 2011; Rotheram-Borus, 2001; Thastum, 2006) and parent-child relationships (Rochat, 2017). However, we should be cautious about attributing positive outcomes solely to improved parent-child communication, as other elements of the treatment—such as psychoeducation and professional support in preparing and reviewing the parent-child conversations within the Family Talk Intervention—may also have contributed. Overall, existing interventions aimed at enhancing parent-child communication about parental illness generally show positive results. Our findings, particularly in Subtheme 4.1, suggest that challenges may arise from “incomplete or insensitive communication,” which can lead to “partial comprehension or misconceptions” in children. Therefore, it is crucial to develop interventions tailored to the specific needs of parents with BD and their children, working closely with families to support effective and sensitive communication.

To develop a sensitive and suitable intervention for families affected by parental BD, it is crucial to address the unique communication challenges in this context. First, comparing this study with literature on life-threatening physical diseases reveals distinct difficulties in communicating about severe mental illnesses with children. These illnesses often manifest through emotions and behaviours, blurring the distinction between the illness and personal identity (**Subtheme 3.3**). This can lead individuals to feel overly responsible for the consequences of their illness, potentially fostering guilt and self-doubt, as seen in previous studies with parents with BD (Anke 2019; Davison 2018; Wilson 2009). Additionally, societal perceptions may lead others to hold parents with BD accountable for their condition, contributing to stigma and mistrust (Subtheme 2.2). Similar issues of guilt and external judgement are noted in parent-child communication studies on other parental disorders like unipolar depression (Pihkala 2008), psychosis (Strand 2020), and substance abuse (Pihkala 2017). Next, combining this study with our previous meta-synthesis (Tu et al. 2025), we noted that BD may involve greater unpredictability and inconsistency in parenting practices than other mental illnesses, likely making it difficult to separate the illness from the parent’s identity, thereby complicating understanding and communication. In conclusion, effective communication about parental BD involves helping children differentiate between the illness and the parent, maintaining love and trust and supporting the restoration of the parental role. Flexibility and customisation in the timing, setting, participants, and materials used in communication are necessary to accommodate BD’s fast-changing nature.

Our comparative analysis integrates parents’ lived experiences and stakeholders’ systemic insights to explore how families discuss parental BD with children. By situating our findings within broader literature on communicating parental mental illnesses to children—including interviews with parents across various diagnoses (Rapa et al. 2024), non-affected partners (Ballal and Navaneetham 2018), and adult mental healthcare professionals (Dalton et al. 2024)—we highlight experiences tied to BD’s episodic and mood-driven nature that may be particularly prevalent in BD families. Compared to Rapa et al. (2024), parents in our study emphasised reassuring their children of their consistent love and that they will return to being the parent they usually are during BD episodes, and preparing them for future episodes during stable periods. In contrast to Ballal and Navaneetham (2018), non-BD partners in this study noted that abrupt mood shifts in parents with BD can challenge children’s abilities to process and adapt to parental BD, compounded by the ill parents’ avoidance of discussing their condition, often leaving the non-affected partner solely responsible for communication, heightening their emotional burden. While Dalton et al. (2024) also underscored professional involvement and coordination, stakeholders in this study highlighted communication difficulties when both the parent and the child experience mental health challenges and the loneliness children may feel due to the unique and often hard-to-explain dynamics of living with a parent with BD, observations which are less evident in studies of broader mental illnesses (Chen et al. 2021, 2023; Rapa et al. 2024).

The insights from this study should be considered in light of its limitations. The predominantly well-educated, co-parenting, white participants from South England limit the generalisability of the findings to more diverse educational, socio-economic, racial, and regional groups across the UK. Additionally, all the participating parents had a child in a specific age group who had experienced anxiety issues, which may limit the applicability of the findings to families without significant psychological problems in children. It also raises questions about how these issues might evolve during adolescence and the transition to adulthood. The reliance on self-reported data may also introduce recall bias, as some participants reflected on experiences with older children, potentially skewing the accuracy of recalled interactions. However, this retrospective perspective can also provide deeper insights into past parenting practices. A notable limitation is the exclusion of children’s perspectives, as the study gathered insights solely from parents and support providers, without triangulating these perspectives with children’s views. This could potentially overlook critical nuances in family dynamics, particularly differing perceptions between parents and children (Strand 2020). Furthermore, the study population and thematic structure may be biased toward families who are already openly about BD, as eligibility required the willingness and ability to share BD-related parenting experiences. This may over-represent participants who are comfortable with disclosure, potentially underestimating the challenges for those who are less inclined or able to communicate about BD. These limitations underscore the need for more inclusive research that captures a broader familial perspectives and readiness levels to fully understand the complexities of communicating about BD.

Future research could address these limitations by exploring the experiences of diverse family structures and cultural backgrounds in relation to discussing parental BD with children, while directly involving children’s viewpoints for a more comprehensive understanding of BD’s familial impact. It could also delve deeper into the roles of non-BD partners and professionals in this communication process and explore the challenges they face. Furthermore, developing tailored interventions that meet the needs of parents with BD, their partners, and children will be crucial in enhancing familial communication. Such efforts should inform policy initiatives to improve primary healthcare sensitivity and support mechanisms for families dealing with BD, ensuring that communication needs are met comprehensively and effectively across all levels of care. Additionally, addressing the disconnect between adult and child mental health care systems is vital, as this gap can hinder effective support for parent-child communication about BD. Strengthening the integration of these services could significantly enhance support frameworks and ensure a more coordinated approach to managing BD within family settings.

## Conclusions

This study emphasises key elements and principles in supporting parent-child communication about parental BD. Effective communication of parental mental health diagnoses has been shown to foster understanding, improve relationships, and enhance children’s mental health. Future research should explore perspectives of children and a broader range of family dynamics to deepen our understanding of these interactions. These findings should inform interventions that support parent-child communication in families dealing with parental BD and equip professionals to better identify and address the communication needs of these families.

## Electronic supplementary material

Below is the link to the electronic supplementary material.

Supplementary Material 1

## Data Availability

No datasets were generated or analysed during the current study.

## Abbreviations

BD: Bipolar Disorder
FTI: Family Talk Intervention
PPI: Public and Patient Involvement
