# Supplementary material for: Communication with children about parental bipolar disorder: a qualitative interview study
Source: Int J Bipolar Disord. 2025 May 24;13:19. doi: 10.1186/s40345-025-00384-9 (PMC12103402; doi:10.1186/s40345-025-00384-9)
Supplement: Supplementary file 1 — Supplementary Material 1 [file 40345_2025_384_MOESM1_ESM.docx]

**Supplement 1: Interview Topic Guide**

| **Part 1: Parenting Experiences in the Context of Parental BD**  Can you tell me about yourself and your family?   - How many people are in your family? What would you like me to call them?   How would you describe your child/ren?   - What is their character like? - How is their school life? - What are their interests or hobbies? - How would you describe your relationship with your child/ren?   Can you give me a brief overview of your parenting experiences?   - How do you and the other caregiver divide up childcare responsibilities? What led to this arrangement? - Do you and the other caregiver have different parenting styles? If so, how would you describe the differences?   Can you describe any problems or challenges you have faced in parenting?   - What are the most challenging aspects for you? - How do you try to resolve these challenges? - What resources or strategies have you found helpful?   Can you provide an overview of your mental health condition?   - How is your bipolar disorder managed? For example, are you on medication or receiving psychotherapy? - What medication are you taking? - Many people with bipolar disorder experience mood changes from high to low. Is that true for you? How do you feel when you are in a low mood or high mood? - How do these mood changes affect your life and your relationships with others?   Do you notice a difference in your parenting when you are feeling high or low?   - Do you think your emotions affect your relationship with your child? How? - Have you ever had to go to the hospital due to your illness? What was your child’s understanding of the situation? How did your child react, and was there any impact on them? Did you explain the situation to them? - Do you think medication has influenced how you parent? If so, how? - Do you think societal attitudes toward bipolar disorder have affected your role as a parent or impacted your child? |
| --- |
| **Part 2: Parenting Experiences Dealing with Child Anxiety in the Context of Parental BD**  Has your child experienced difficulties with anxiety, fears, or worries?   - When did this start? Is it still happening, or has it resolved? - Can you provide one or two specific examples of your child’s anxiety? - Can you describe the anxiety in more detail? - Are there moments or situations when your child’s anxiety becomes better or worse? - What is your child anxious or worried about? Has your child shared what they are anxious about?   What do you think has influenced your child’s anxiety?   - Do you think your child was born with it, or has it been influenced by specific experiences?   When your child feels unwell due to anxiety (or is unwilling to do something), how do you deal with it?   - What do you say to or do for them? - What are the most challenging aspects for you? - How have you tried to resolve these challenges? What things have you found helpful? - Has anyone provided support or help (e.g., school, charity, NHS)? In what way?   What effects have your child’s anxiety problems had on you (e.g., emotions or daily life)?  Do you feel your emotions have affected how you manage your child’s anxiety, either in a positive or negative way? |
| **Part 3: Parenting Experiences Disclosing Illness in the Context of Parental BD**  Have you ever tried to discuss your emotional difficulties or diagnosis with your child?  If Yes: How did you explain your difficulties to your child?   - Do you think discussing your diagnosis affected your parenting or the parent-child relationship? In a positive or negative way? - Do you think discussing your diagnosis had an impact on your child’s anxiety? In a positive or negative way? - Do you have any specific concerns about how sharing your diagnosis or difficulties might influence your child?   If No: What are your thoughts on discussing your diagnosis with your child?   - Do you have any particular concerns about the possible effects of sharing your diagnosis or difficulties with your child? |
| **Part 4: Parenting Experiences Disclosing Illness in the Context of Parental BD**  What do parents with bipolar disorder (or their partner/other caregivers) need to help their children with anxiety problems?   - Key elements of intervention: Why do you think those elements would be helpful? - Barriers to intervention: What do you think could be difficult or problematic about that?   How should this support be provided?   - Do you see any advantages or disadvantages to receiving support online compared to in person? - Do you think there are any advantages or disadvantages to receiving this support in a group setting with other parents, compared to a one-to-one approach?   What do you think about supporting parents to help their children overcome anxiety in families affected by bipolar disorder?   - What concerns, if any, do you have about this type of treatment? - Do you think there are any reasons why it would be difficult for you to benefit from this kind of treatment?  Can you share any similar experiences or positive outcomes related to bipolar disorder in your family? Is there anything else you’d like to add, or any final thoughts or follow-up questions that we haven’t covered yet? |

**Supplement 2: Frequency of Coded References for Each Theme and Subtheme by Participant Group**

|  | **T1: Perceived Advantages** | | **T2: Perceived Challenges** | | | **T3: Key Elements** | | | | **T4: Practical Strategies** | | |
| --- | --- | --- | --- | --- | --- | --- | --- | --- | --- | --- | --- | --- |
| **Types** | **1.1** | **1.2** | **2.1** | **2.2** | **2.3** | **3.1** | **3.2** | **3.3** | **3.4** | **4.1** | **4.2** | **4.3** |
| Fathers with BD  (n=3) | 3 | 2 | 2 | 0 | 5 | 5 | 2 | 2 | 3 | 4 | 3 | 1 |
| Mothers with BD  (n=5) | 4 | 3 | 2 | 3 | 2 | 8 | 1 | 2 | 2 | 4 | 4 | 0 |
| Non-bipolar Parents  (n=3) | 1 | 0 | 0 | 2 | 1 | 10 | 5 | 5 | 3 | 5 | 6 | 1 |
| Mental health Professionals  (n=7) | 4 | 1 | 2 | 3 | 3 | 9 | 3 | 3 | 0 | 6 | 7 | 6 |
| Charity Workers  (n=5) | 3 | 4 | 3 | 3 | 3 | 9 | 3 | 3 | 3 | 5 | 10 | 3 |
| Number of Codes | 6 | 4 | 4 | 4 | 3 | 14 | 5 | 6 | 4 | 8 | 10 | 6 |
| ^1^ Multiple quotes from one participant group within a code are counted only once | | | | | | | | | | | | |

**Supplement 3: Related Resources**

- **Women and Bipolar Webinars - "Parenting with Bipolar Webinar" (Bipolar UK)**
  Hosted by Dr. Clare Dolman, this webinar explores the challenges and strategies of parenting with bipolar, featuring insights on communicating the condition with children.

https://www.bipolaruk.org/Blog/women-and-bipolar-webinar-catch-up#parenting

- **FAMpod Website - "Parent Talk" and "TeenTalk" Resources**
  FAMpod offers resources for families and clinicians. "Parent Talk" provides information on depression and resilience for parents and children, while "TeenTalk" is designed for teens, discussing depression in a teen-friendly format. Clinicians can also explore the Family Talk course for implementing the Family Talk intervention.

https://fampod.org/
